# Supplementary material for: Clinical outcomes and inflammatory marker levels in patients with Covid-19 and obesity at an inner-city safety net hospital
Source: PLoS One. 2020 Dec 16;15(12):e0243888. doi: 10.1371/journal.pone.0243888 (PMC7744045; doi:10.1371/journal.pone.0243888)
Supplement: S1 Table — CRP = C reactive protein, LDH = lactate dehydrogenase ALC = absolute lymphocyte count. (PDF) [file pone.0243888.s001.pdf]

Table S1. Median (range) inflammatory markers and absolute lymphocyte count on hospital days 1 and 2 as well as maximum FiO2 during hospitalization by body mass index (BMI) group.

	BMI < 25<br>N=186	BMI 25-29.9<br>N=235	BMI 30-34.9<br>N=182	BMI 35-39.9<br>N=98	BMI > 40<br>N=80	p-value
Day 1 CRP (mg/L)	67.9 (0.4-447.7)	94.6 (0.7-683.3)	73.0 (0.3-429.5)	73.5 (0.4-421.4)	64.1 (3.7-381.5)	0.060
Day 2 CRP (mg/L)	67.2 (0.6-460.0)	98.7 (0.3 – 600.7)	84.7 (0.2-378.7)	74.8 (0.4-408.5)	68.3 (3.5-475.5)	0.056
Day 1 LDH (U/L)	311 (100-2824)	359 (154-967)	361 (47-2897)	387 (167-921)	370 (153-1485)	0.0027
Day 2 LDH (U/L)	297 (141-2643)	352 (145 – 1296)	345.5 (149-2507)	338 (175 – 879)	347.5 (153 – 1059)	0.0005
Day 1 Ferritin (ng/mL)	594 (26-33,511)	645.5 (12-33,511)	519 (12 – 33,511)	311 (25 – 8135)	421 (9 – 13263)	0.0002
Day 2 Ferritin (ng/mL)	683 (29-33,511)	671 (13 – 1465)	519.5 (12-33,511)	324.5 (12- 6,174)	413 (13 – 10,345)	<0.0001
Day 1 D-dimer (µg/mL)	382.5 (150- 44,123)	335 (150 – 16,704)	300 (150 – 54,550)	296 (150 – 48,048)	263 (150 – 8,206)	0.0249
Day 2 D-dimer (µg/mL)	403 (150 – 10,779)	346 (150 – 14,027)	331 (150 – 15,840)	312 (150 – 15,840)	245 (150 – 51,299)	0.0047
Day 1 ALC (1000/µL)	1.1 (0.2-4.0)	1.1 (0.3-5.1)	1.1 (0.3-62.3)	1.3 (0.3-5.2)	1.2 (0.2-3)	0.0013
Day 2 ALC (1000/µL)	1.2 (0.1 – 101.0)	1.1 (0.3 – 9.2)	1.2 (0.1 – 51.2)	1.4 (0.5 – 4.3)	1.3 (0.2 – 3.2)	0.0285
Max FiO2	27% (21-100)	30% (21-100)	30% (21-100)	30% (21-100)	35.3% (21-100)	0.15

CRP = C reactive protein, LDH = lactate dehydrogenase ALC = absolute lymphocyte count
